# 肺癌肉瘤1例

**DOI:** 10.3779/j.issn.1009-3419.2011.05.16

**Published:** 2011-05-20

**Authors:** 贤雨 张, 丽英 班, 洁 张

**Affiliations:** 116011 大连，大连医科大学第一附属医院肿瘤科 Department of Oncology, the First Afliated Hospital, Dalian Medical University, Dalian 116011, China

癌肉瘤（carcinosarcoma, CS）是一种含恶性上皮成分及异源性恶性间叶成分如骨、软骨、骨骼肌成分的复合型恶性肿瘤，多见于子宫，亦可见于鼻咽部、乳腺、支气管、膀胱及食道等处。原发性肺癌肉瘤罕见，约占肺部恶性肿瘤的0.2%-0.3%^[1]^。本文结合文献报告1例肺癌肉瘤，以对其临床症状、病理特征、影像学特点、治疗及预后有所了解。

## 1 病例资料

男性，49岁，因右侧颈肩疼痛3个月，渐加重半个月，咳嗽1周于2009年11月27日入院。主诉有右侧颈肩疼痛伴前胸疼痛、咳嗽、咳痰，无痰中带血及咯血，活动时有全身神经放射性疼痛，双脚、小腿麻木，走路不稳。2009年11月25日曾在我院查胸部CT（图 1A、图 1B）示两肺弥漫性大小不等的磨玻璃样高密度影，部分内见钙化灶，右肺下叶可见一不规则肿块影，其内清晰支气管影，管壁钙化，右侧斜裂可见钙化灶；右肩胛骨可见骨质破坏，周围软组织见多发斑片状高密度影。查体：右肩胛区膨隆，压痛不明显。双肺可闻及干啰音，剑突平面以下痛觉、温觉迟钝，触觉正常，四肢肌力5级，双膝反射亢进，双侧Babinski征阴性。吸烟20年，20支/天。

**1:**
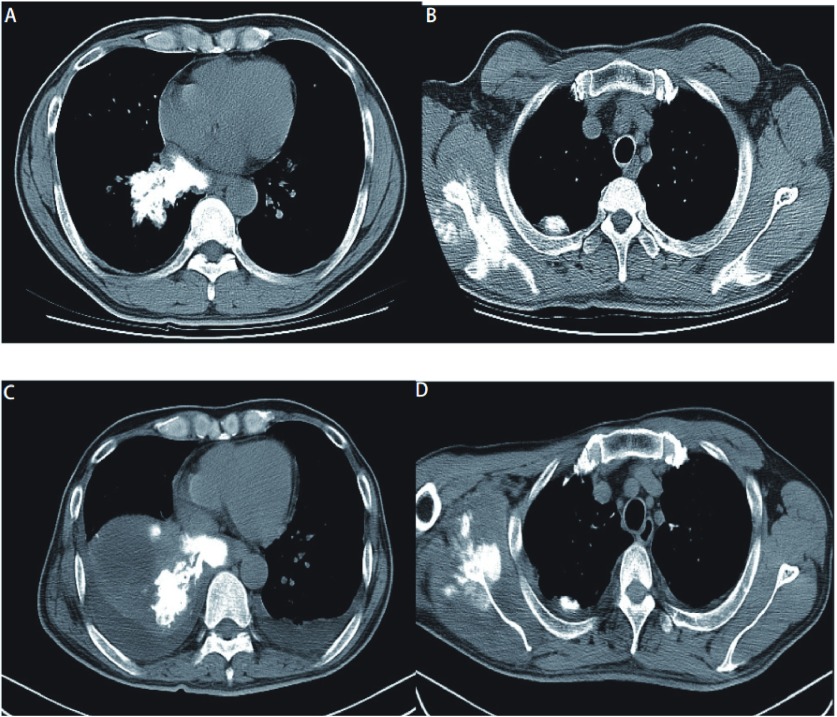
患者治疗前后胸部CT。A：右肺下叶一不规则肿块影，其内清晰支气管影，管壁钙化；B：右肩胛骨可见骨质破坏，周围软组织见多发斑片状高密度影；C、D：治疗后，右肺下叶肿块影较前变大，并出现双侧胸腔积液 The chest computed tomography (CT) before chemotherapy and after chemotherapy. A: CT showing an irregular tumor in the inferior right lobe, in which we can see the clear bronchitis shadow and calcification of tubal wall; B: CT showing the destruction of the right scapula and patching high density shadow in surrounding soft tissue; C, D: after the chemotherapy, showing an enlarging tumor in the in ferior right lobe and bilateral pleural effusion

入院后行纤支镜检查，镜下见右下后基底可见粘膜向腔内隆起，取组织及刷擦涂片送检癌细胞、TB、TB-PCR；内基底支管口狭小，刷擦涂片送检，余支气管正常。灌洗液检查见低分化非小细胞恶性肿瘤（图 2A）；支气管粘膜病理显示支气管活检物少许恶性肿瘤组织形态呈肉瘤样，伴有成骨及成软骨，符合癌肉瘤（图 2B）。由于病理取材太少，未能行免疫组化检测。入院后第3天出现咯血，第5天出现双下肢肌力减退，肌张力增高。2009年12月4日行头部CT检查显示轻度脑白质脱髓鞘改变；胸椎MR显示胸4、胸5病变，考虑转移，脊髓受压。临床诊断为肺癌肉瘤Ⅳ期，双肺、骨转移，肺内感染，截瘫。因无手术指征于2009年12月11日予多西他赛+顺铂行第1周期化疗，同时予伊班膦酸治疗骨转移，硫酸吗啡缓释片止痛治疗，病情曾一度好转。2010年1月6日复查胸部CT显示右肺下叶可见一不规则肿块影，大小约9.51 cm×5.72 cm，较前（2009年11月25日胸片）变大，其内可见包绕的支气管影与团片状钙化灶；双侧胸腔积液（图 1C、图 1D）。疗效评价为进展。患者反复肺部感染，予相关治疗无好转，于2010年1月15日出现呼吸衰竭而死亡。

**2:**
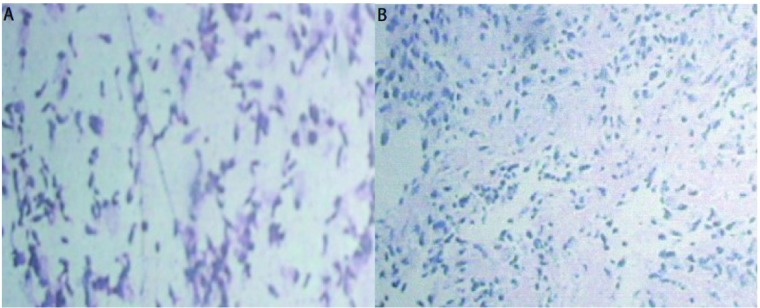
病理检查结果。A：低分化非小细胞恶性肿瘤（HE, ×100）；B：少许恶性肿瘤组织形态呈肉瘤样，伴有成骨及成软骨（HE, ×40） Microscopic sections. A: Microscopic sections showing a poorly differentiated non - small ce ll malignant tumor (HE, A×100); B : Histology of the tumor demonstrated heterologous elements such as cartilage and bone (HE, ×40)

## 2 讨论

### 2.1 组织来源

CS在1981年WHO对肺肿瘤的分类中，被定义为一种恶性上皮成分及恶性间叶成分的复合型恶性肿瘤。1999年WHO的新分类^[2]^中明确提出，肺肉瘤样癌（pulmonary sarcocarcinoma, PSC）中的恶性间叶成分应为异源性的，肿瘤中恶性的骨、软骨、骨骼肌成分是诊断PCS的重要依据。2004年世界卫生组织肺肿瘤组织学分类^[3]^中将PSC定义为一组含有肉瘤形态细胞或肉瘤样分化的非小细胞肺癌（non-small cell lung cancer, NSCLC），具有多形性癌、梭形细胞癌、巨细胞癌、癌肉瘤和肺母细胞瘤5种亚型^[4]^。

目前关于CS的组织发病机理还不清楚，随着分子生物学技术的发展越来越多的学者支持单克隆学说^[5]^，即CS是有一种多能干细胞向上皮和间质方向分别分化的结果，这种情况下两种肿瘤的组织成分互相混合，融合一体，这种类型被称为“复合瘤”或“联合瘤”。

### 2.2 病理分析

PCS可以是任何类型的癌与肉瘤以任何比例混合而成。癌的成分以鳞癌最多见，另外还可见腺癌、肺泡细胞癌、肺大细胞癌或小细胞癌等。肉瘤成分以纤维肉瘤最多见，还可有平滑肌肉瘤、横纹肌肉瘤、软骨肉瘤、骨肉瘤等。癌和肉瘤成分都可以是一种或几种混合存在。本例行纤支镜检查时，于灌洗液中发现低分化非小细胞恶性肿瘤，于支气管粘膜取组织行病理检测发现少许恶性肿瘤组织形态呈肉瘤样，伴有成骨及成软骨，符合癌肉瘤诊断标准，但由于组织提取较少，未能进一步行免疫组化检测。

### 2.3 影像学分析

PCS的CT报道少见，可分为周围型及中心型，以周围型多见^[6]^，肿块直径 > 5 cm者占60%以上，而肺癌或肺炎性假瘤直径多 < 5 cm，右肺略多于左肺。中央型肺癌阻塞支气管时可出现阻塞性肺炎及肺不张。周围型肺癌边缘可见分叶及毛刺征，肿块密度因其组织成分不同变化较大，可出现坏死、空洞及钙化，周边可侵犯胸膜引起胸膜凹陷。CT值多偏低（30 HU左右）^[6]^，轻度强化。本例行CT检查时也发现右肺下叶肺门下方一不规则肿块影，直径 > 5 cm，其内可见包绕的支气管影及团片状钙化灶。

CT诊断PCS缺乏特异性，而^18^F-FDG PET和^18^F-FDG PET/CT在肉瘤的病理学分级、良恶性鉴别、临床分期及再分期、局部复发评估和疗效监测等方面均发挥出较大的优势^[7]^。

### 2.4 临床分析

PCS好发于50岁以上的患者，男性发病多于女性，比例为2.12:1-6:1^[8]^。PCS多以咳嗽、咳血、胸闷、发热等呼吸系统症状为起病症状，少数也表现为肩痛、声音嘶哑、杵状指等肺外症状。若肿瘤位于周围可有胸痛或胸腔积液。其临床表现与其它类型肺癌相比缺乏特异性，临床上不易鉴别。90%的患者有重度吸烟史，早期常被误诊为肺结核，而晚期常被误诊为肺癌。此外患者可有结核病史。本例患者发病时年龄仅为49岁，有20年吸烟史，主要以肩痛、咳嗽起病，于治疗后期出现胸腔积液。

### 2.5 治疗与预后

PCS对放疗和化疗都不敏感，手术成为首选的治疗方式。由于原发性肺癌肉瘤的体积较大、恶性程度高，手术方式应以根治性切除为主，尤其在早期手术治疗的预后较好。对于不能手术或术后患者，辅以放疗、化疗或生物治疗，能够延长患者的生存时间。由于目前尚无标准和满意的治疗方案，化疗方案多参照NSCLC化疗方案^[9]^。但是有学者认为PCS的预后可能与肿瘤内的肉瘤成分有关，因此适用于软组织肉瘤的综合治疗同样适用于PCS的治疗。

比较PSC和NSCLC患者预后的研究^[10]^显示，PSC复发风险较高、预后较差，并指出辅助放疗、化疗适用于肿块大、有胸壁浸润及淋巴结转移的患者，因为这类患者术后复发风险高。Mochizuki等^[11]^得出同样的结论，并且发现病理分期、纵隔淋巴结转移及肿块出血坏死是影响患者无病生存期的重要因素。

周琪等^[8]^研究分析了64例PCS患者，这些患者行全肺切除、肺叶切除及姑息性手术，并且术后辅以MVP（丝裂霉素+长春花碱+顺铂）、MAP（丝裂霉素+多柔比星+顺铂）、NP（长春瑞滨+顺铂）方案化疗，随访发现全肺切除术组患者预后较好（*P*=0.036），化疗组的5年生存率稍高于未化疗组，但无统计学差异（*P*=0.971）。Hong等^[12]^对12例（其中9例接受了肿瘤根治术后进展，3例晚期患者未施行手术）晚期PSC患者进行盐酸吉西他滨+顺铂的姑息化疗，结果显示7例出现疾病进展，3例病情稳定，仅有2例患者为部分缓解，以化疗开始计算的中位生存时间仅为8个月。本例患者诊断时已为晚期，无法行根治性切除术，给予姑息性化疗及对症治疗。化疗方案的选择参照NSCLC，即含铂类的两药联合（顺铂+多西他赛）。患者于首周期化疗后临床症状虽有短暂的缓解，但因病情进展迅速，于治疗后1个月即死亡。

PCS为一种罕见的肺部恶性肿瘤，预后较NSCLC差，在治疗方法的选择上不能单纯参照NSCLC，需要更多的临床研究来寻找适合这类肿瘤的综合治疗措施，以改善患者的生活质量，延长生存期。
